# Maspin impairs the function of endothelial cells: an implying pathway of preeclampsia

**DOI:** 10.1186/s12884-017-1525-z

**Published:** 2017-09-29

**Authors:** Ying Zhang, Hao Liu, Xinwei Shi, Fuyuan Qiao, Wanjiang Zeng, Ling Feng, Dongrui Deng, Haiyi Liu, Yuanyuan Wu

**Affiliations:** 1 Department of Obstetrics and Gynecology, Tongji Hospital, Tongji Medical College, Huazhong University of Science and Technology, Wuhan, Hubei China; 2 Department of Urology, Wuhan Third Hospital, Guanggu on campus, Wuhan, Hubei China

**Keywords:** HUVECs, Maspin, Hypoxia, Angiogenesis, Preeclampsia

## Abstract

**Backgroud:**

Widespread endothelial injury contributes to the occurrence of preeclampsia. Maspin, first identified as a tumor suppressor, plays a critical role in cell invasion and angiogenesis. Our previous studies found that the expression of maspin was increased in preeclampsic placenta. In this research, we studied the function of human umbilical vein endothelial cells (HUVECs) to explore the role and possible mechanism of maspin gene in the pathogenesis of preeclampsia.

**Methods:**

HUVECs were treated with different concentration of recombinant human maspin protein (r-maspin) during normoxia and hypoxia, we detected the proliferation, apoptosis, migration and tube formation of HUVECs. We also assessed nitride oxide (NO) synthesis and the expression of matrix metalloproteinase 2 (MMP2) to further explore the underlying molecular mechanism.

**Results:**

There was only slight maspin expression at mRNA level in HUVECs. Treated HUVECs with r-maspin, the proliferation of HUVECs was significantly promoted both under normoxia and hypoxia. The tubes formed by HUVECs were significantly inhibited and NO synthesis was significantly reduced by r-maspin. Meantime, r-maspin also inhibited MMP2 expression and activity in HUVECs. However, there was no significant change in the migration and apoptosis of HUVECs.

**Conclusions:**

Maspin may be an important participant for mediating endothelial function and ultimately leads to the occurence of preeclamsia.

## Background

Preeclampsia (PE) is a pregnancy disorder that is characterized by the onset of hypertension and proteinuria in previously normotensive women after the twentieth week of gestation [1]. Acute renal failure and long-term cardiovascular morbidity can occur in patients with severe preeclampsia [1]. The clinical symptoms of preeclampsia are relieved rapidly after the delivery of placenta [2]. It is generally considered that widespread endothelial injuries contribute to the occurrence of preeclampsia [3].

Mammary serine protease inhibitor (maspin) is an epithelial-specific Class II tumor suppressor gene and belongs to the serine protease inhibitor (serpin) superfamily [4]. As a tumor suppressor, maspin has inhibitory effect on the invasion, motility, and metastasis of tumor cells [5–7]. In addition, maspin is also an important inhibitor of angiogenesis. Zhang et al. [8] have first demonstrated that maspin can effectively block neovascularization by the rat cornea pocket model in vivo and inhibit the migration of endothelial cells in vitro. Cher et al. [9] have also shown that the vasculature density is reduced by maspin in a prostate xenograft model.

The development of placenta starts with the successful trophoblast invasion and then follows by the completion of vascular remodeling, which is similar to the process of tumor development that requires tumor cell invasion and tumor angiogenesis [10]. Dokras et al. [11] reported that maspin was differentially expressed in human placenta and plays an important role in regulating the invasive capabilities of cytotrophoblasts throughout gestation. Our previous studies have also shown that the level of maspin expression in preeclampsic placenta is up-regulated [12–14]. Meantime, the level of maspin expression is up-regulated during hypoxia alongside a decrease in invasive abilities of human first-trimester extravillous trophoblast cell line (TEV-1) [12, 13].

Based on these studies, we hypothesize that maspin plays an important role in the occurrence of preeclampsia by impairing the fetoplacental vasculature. We treated human umbilical vein endothelial cells (HUVECs) with different concentration of human recombinant maspin protein (r-maspin) during normoxia and hypoxia. And then we examined the proliferation, apoptosis, migration, tube formation of HUVECs. Meanwhile,in order to further understand the underlying molecular mechanism, we also assessed the nitride oxide (NO) synthesis, the expression and activity of matrix metalloproteinase 2 (MMP2) in HUVECs.

## Methods

### Cell culture

HUVECs were obtained from American Type Culture Collection (ATCC, USA) and we cultured HUVECs with RPMI-1640 (HyClone, USA) supplemented with 10% fetal bovine serum (FBS) (Gibco, USA). We added CoCl_2_ (cobalt dichloride, Sigma-Aldrich, USA) to a final concentration of 300 μmol/L to induce chemical hypoxia [13, 15].

### Real-time PCR

Total RNA was extracted for reverse transcription of cDNA according to the manufacturer’s protocol (TransGen Biotech, Beijing, China). The real-time PCR reaction system contained 10 μL SYBR Green PCR Mix (DBI Bioscience, Germany), 1 μL internal primers and 1 μL cDNA. The conditions were as follows: initial denaturation at 94 °C for 5 min, 40 cycles of 94 °C for 30 s, 60 °C for 30 s, 72 °C for 30 s, and a final extension at 72 °C for 10 min. The primer sequences were listed in Table 1.

**Table 1:** The Related Primers Sequences and Product Size

primers	sequence	length
Maspin (internal primers)	F:5′-CCACAGGCTTGGAGAAGATTGA-3′ R:5′-GGTCAGCATTCAATTCATCCTTGT-3	338 bp
Maspin (external primers)	F:5′-CCAAGGCTTGTCTGGAAAATCTA-3′ R:5′-TTCAATTCATCCTTGTGCTGCAG-3’	196 bp
MMP-2	F:5’-ACATCAAGGGCATTCAGGAGC-3′ R:5′-ACAGTCCGCCAAATGAACCG-3’	181 bp
*β*-ACTIN	F:5’-CACCCAGCACAATGAAGATCAAGAT-3′ R:5′-CCAGTTTTTAAATCCTGAGTCAAGC-3’	317 bp

### Nest PCR

The amplification products of maspin and GAPDH obtained from real-time PCR were used as the substrate for Nest PCR. We performed Nest PCR with 10 μL SYBR Green PCR Mix, 1 μL amplification products, and 1 μL external primers. The conditions were as follows: initial denaturation at 94 °C for 5 min, 30 cycles of 94 °C for 30 s, 54–56.5 °C (with a gradient of 0.5 °C) for 30 s, 72 °C for 30 s, and a final extension at 72 °C for 10 min. The products were resolved on 2% agarose gels (Amresco, USA).

### Western blotting

HUVECs were lysed in RIPA lysis buffer (Beyotime, Jiangsu, China), the cellular debris were centrifuged at 12,000 rpm at 4 °C for 10 min. The supernatant was used for western blotting analysis. We transferred equal amounts of protein extract that were separated by SDS-PAGE (10%) onto polyvinylidene fluoride (PVDF) membranes and then probed with primary antibodies of maspin (Abgent, USA) and MMP2 (BD Pharmingen, San Jose, CA, USA) both at a dilution of 1:100. After incubation with primary antibodies at 4 °C overnight, the blots were probed with horseradish peroxidase-conjugated secondary antibody (Abgent, USA) at a 1:3000 dilution for 30 min at room temperature. The signals were detected by Immobilon reagent (Millipore, Billerica, MA) and visualized by image analyzer (Bio-Rad, Hercules, CA, USA).

### Groups

We treated HUVECs with three different concentration of r-maspin (Peprotech, USA) (M1: 10 ng/ml; M2: 10^2^ ng/ml; M3: 10^3^ ng/ml) under normoxia and hypoxia respectively. PBS was used as a control.

### Proliferation assay

HUVECs were seeded into 96-well plates at a density of 1 × 10^4^ cells/well and cultured for 24 h. Replaced fresh culture medium containing r-maspin and cultured for 6, 12, 24, 48 and 72 h under normoxia and hypoxia respectively. 10 μL CCK8 reagent (Japan Co., Ltd.) were added into each well and mixed gently. After incubation at 37 °C for 1 h, the optical density values (OD) were measured at 450 nm (OD_450_), which represented the number of live cells of each well.

### Apoptosis assay

HUVECs were seeded into 6-well plates at a density of 5 × 10^4^ cells per well and cultured for 24 h. HUVECs were washed and cultured for 24 h and 48 h with fresh culture medium containing r-maspin under normoxia and hypoxia respectively. Annexin V-FITC Apoptosis Detection Kit (KeyGEN, Nanjing, China) was used according to the manufacturer’s protocol. The apoptosis rate was detected by the flow cytometer (BD Biosciences, USA).

### Scratch assay

4 × 10^4^ cells were seeded into 12-well plates and cultured with fresh medium containing 2% FBS for 24 h. Drew a straight line with a width of 0.2 mm at the bottom of each well,washed and captured the images by microscopy (Olympus, Japan). Refreshed the culture medium containing 2% FBS with r-maspin under normoxia and hypoxia for 24 h and captured the images by microscopy (Olympus, Japan).

### Tube formation assay

Cells were seeded into 6-well plates at a density of 5 × 10^4^ cells per well and cultured for 24 h. HUVECs were washed and cultured with fresh medium containing r-maspin under normoxia and hypoxia for another 24 h. HUVECs were harvested for cell suspension at a density of 5 × 10^4^ cells/ml. 300 μL of uniformly dissolved matrigel (BD Biosciences) was added into each well (pre-cooling on ice) and dried for 30 min at 37 °C. Finally, 500 μL cell suspension was added onto the dried matrigel and cultured for 24 h at 37 °C. Images of each group were captured by microscopy (Olympus, Japan) (100× field). and the number of complete tubes of each well was counted under the microscope.

### NO assay

HUVECs were seeded into 24-well plates at a density of 1 × 10^6^ cells per well and cultured for 24 h. Cells were washed and cultured with fresh medium containing r-maspin under normoxia and hypoxia for another 72 h. The supernatant was collected from each group. A NO assay kit (Jiancheng, Nanjing China) was used according to the manufacturer’s protocol.

### Zymography

HUVECs were lysed and the cellular debris were centrifuged. The supernatant was used for electrophoresis with 10% SDS-PAGE containing 0.1% gelatin (Sigma, USA) at a constant voltage of 165 V until the tracking dye reached the bottom of the gel. Put the gels into elution solution (containing 2.5% Triton X-100, 50 mM Tris-HCl, 5 mM CaCl_2_, 1 μM ZnCl_2_) for 60 min. The gel was washed for 30 min, incubated at 37 °Cfor 18 h, stained with 0.05% Coomassie Brilliant Blue for 20 min and visualized by image analyzer (Bio-Rad, Hercules, CA, USA).

### Statistical analysis

The statistical software SPSS 16.0 (SPSS Inc., Chicago, IL, USA) was used for data analysis. All data are presented as the mean ± SEM and the results were submitted to statistical analysis using one-way ANOVA. A *P*-value of <0.05 was considered statistically significant.

## Results

### Expression of maspin in HUVECs

**Fig. 1:**
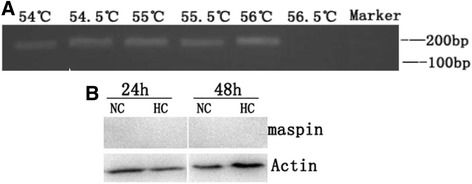
Maspin expression in HUVECs. **a** The level of maspin mRNA expression was determined by 2% agarose electrophoresis of the amplification products from Nest PCR using different annealing temperatures. **b** The level of maspin protein in HUVECs during normoxia (NC) and hypoxia (HC). β-actin was used as the control

We observed a band by the 2% agarose gel electrophoresis with the Nest PCR products except at the annealing temperature of 56.5 °C (Fig. 1a). We couldn’t detect the protein expression of maspin in HUVECs that were cultured for 24 h and 48 h (Fig. 1b). These results indicated that there was only slight maspin expression at mRNA level in HUVECs.

### R-maspin protein significantly promoted HUVEC proliferation

**Fig. 2:**
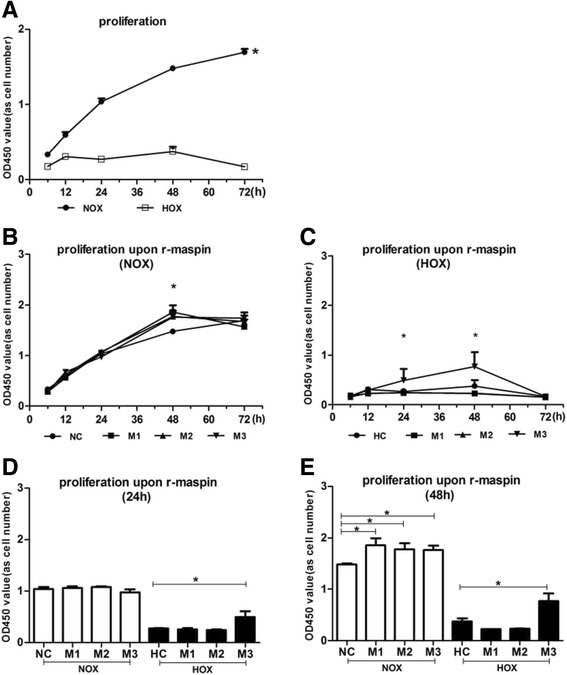
The effect of r-maspin on HUVEC proliferation. **a** HUVEC proliferation during normoxia and hypoxia. **b** and **c** The proliferation of HUVECs treated with different concentration of r-maspin during normoxia (**b**) and hypoxia (**c**). **d** and **e** Proliferation of HUVECs cultured for 24 h (**d**) and 48 h (**e**) with r-maspin. *P < 0.05. M1: 10 ng/mL; M2: 10^2^ ng/mL; M3: 10^3^ ng/mL

We observed a continuously proliferation trend of HUVECs for 72 h under normoxia (Fig. 2a). Nevertheless, the proliferation was significantly inhibited under hypoxia (Fig. 2a). The proliferation rates of HUVECs treated with r-maspin under normoxia was nearly 1.25-fold than that in the normal control and the effect was especially dramatic at cultured 48 h (M1: 1.859 ± 0.13 vs 1.478 ± 0.02; M2: 1.774 ± 0.12 vs 1.478 ± 0.02; M3: 1.764 ± 0.09 vs 1.478 ± 0.02, *P* < 0.05) (Fig. 2b and e). Under hypoxia, the proliferative rate of HUVECs with the concentration of M3 at cultured 24 h and 48 h was nearly 2-fold than that of the hypoxia control (cultured 24 h: 0.4910 ± 0.11 vs 0.2723 ± 0.01,cultured 48 h: 0.7698 ± 0.15 vs 0.3740 ± 0.06,*P*<0.05) (Fig. 2c-e).

### R-maspin had no effect on HUVEC apoptosis

**Fig. 3:**
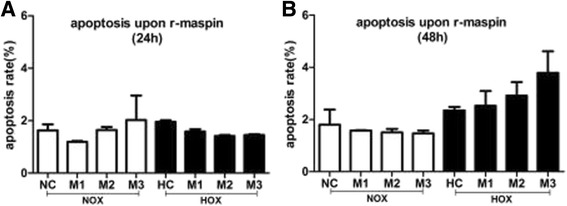
The effect of r-maspin on HUVEC apoptosis. **a** and **b** The rate of apoptosis in HUVECs cultured for 24 h (**a**) and 48 h (**b**) with r-maspin

We analyzed the apoptosis of HUVECs that were treated with r-maspin for 24 h (Fig. 3a) and 48 h (Fig. 3b) under normoxia and hypoxia respectively. Nevertheless, no statistically significant change was observed .

### R-maspin had no effect on the migration of HUVECs

**Fig. 4:**
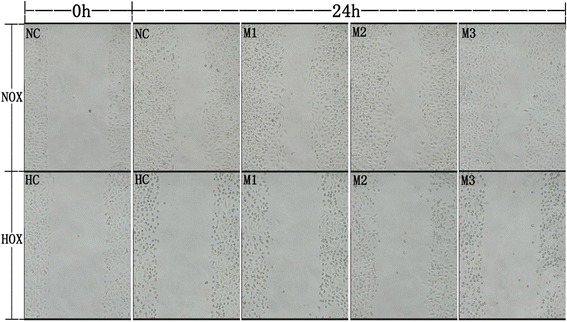
The ability of HUVEC migration with r-maspin. The effect of r-maspin on HUVECs migration during normoxia and hypoxia. NOX:normoxia; HOX: hypoxia

Figure 4 showed that the width of the scratch narrowed in normal control after 24 h while the width of the scratch didn’t change in hypoxic control, which indicated that hypoxia inhibited the migration of HUVECs. There was no obvious change of the width of the scratch in the group of HUVECs treated with r-maspin either under normoxia or hypoxia (Fig. 4).

### R-maspin significantly inhibited HUVEC tube formation

**Fig. 5:**
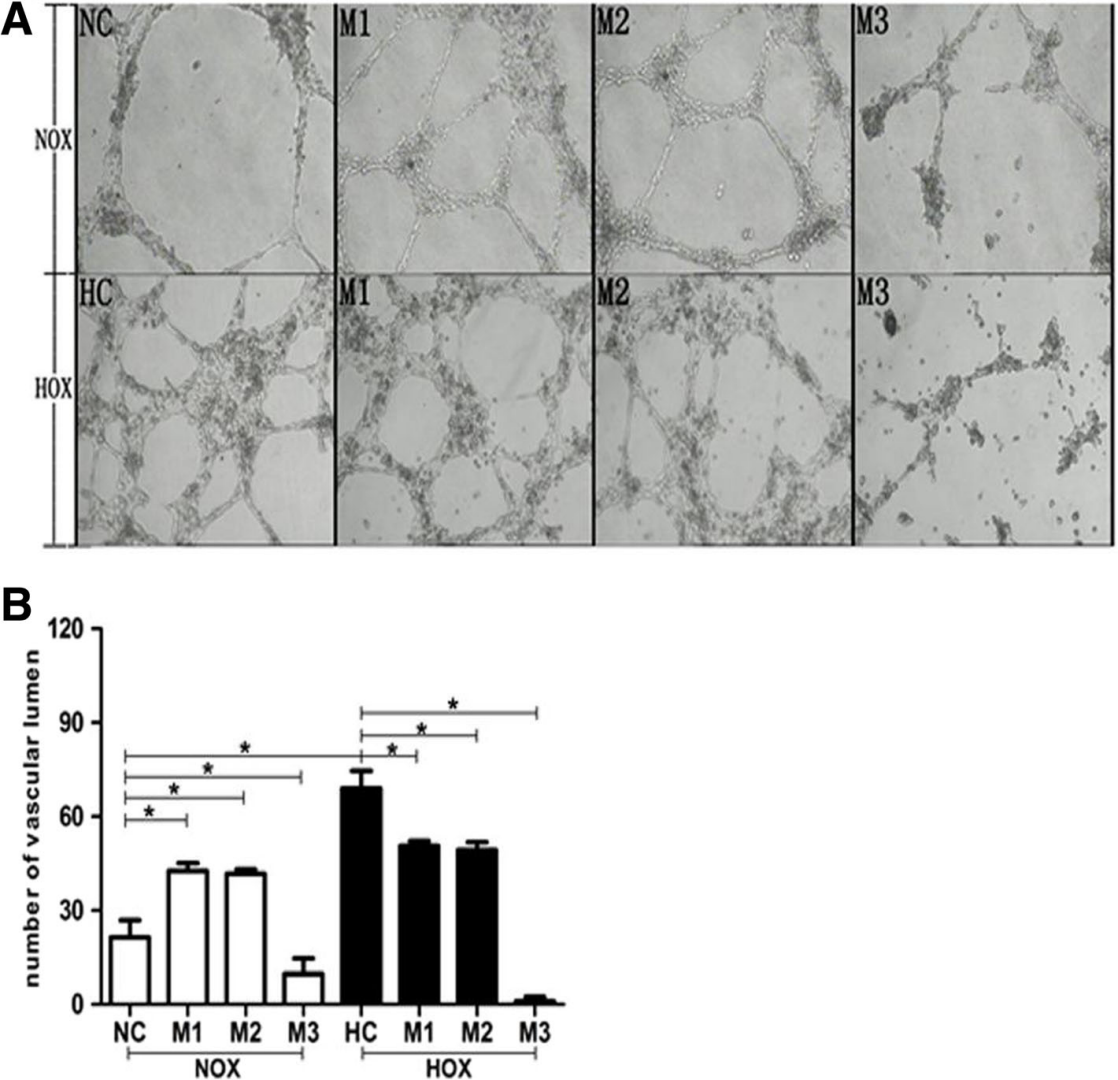
The effect of r-maspin on HUVEC tube formation. **a** The morphology of tubes formed by HUVECs treated with r-maspin (100× magnification). **b** The number of tubes in each group as determined by counting. Each field of vision (40× magnification) was divided into four quadrants with a cross, each quadrant was counted and then summed. Each count was performed in triplicate and then averaged. *P < 0.05

There was a large complete tube that occupied the entire field of view at high magnification (100×) under normoxia (Fig. 5a). However, we observed the aggregation of HUVECs and smaller diameter of tubes formed under hypoxia (Fig. 5a). The number of tubes formed under hypoxia was nearly 3-fold of that in the normal control (Fig. 5b). The diameter of the tubes formed by HUVECs that treated with r-maspin under normoxia reduced by 50% and the number was two times of that in the control group (Fig. 5a, b). Few complete tubes formed by HUVECs with high concentration of r-maspin and we could only observe broken branches. These data implys that the rate of tube formation decreased under normoxia. Similar results were observed in the groups of HUVECs that were treated with r-maspin under hypoxia (Fig. 5a). No obvious change of tube diameter and number were observed with lower concentration of r-maspin (M1) compared with the hypoxic control and few complete tubes formed and only broken branches were observed in the group of HUVECs that were treated with high concentration of r-maspin (M3) (Fig. 5).

### R-maspin inhibited NO synthesis in HUVECs

**Fig. 6:**
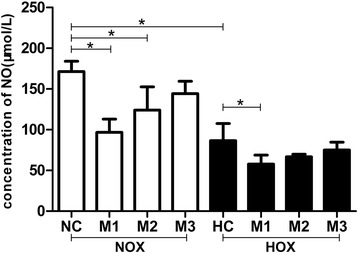
The ability of NO synthesis in HUVECs with r-maspin. NO synthesis in HUVECs treated with different concentration of r-maspin during normoxia and hypoxia. *P < 0.05

NO is a potent vasodilator and it contributes to the maintenance of vascular tone by increasing uterine blood flow in normal pregnancy [16, 17]. The synthesis of NO in HUVECs was reduced by two times under hypoxia of that in normal control (Fig. 6) (84.84 ± 12.32 vs. 171.3 ± 6.332, *P* < 0.01). The NO synthesis of HUVECs treated with different concentration of r-maspin under normoxia significantly reduced than normal control (Fig. 6). There was no statistical difference in the synthesis of nitric oxide in HUVECs treated with r-maspin during normoxia and hypoxia (Fig. 6).

### R-maspin inhibited MMP2 expression and activity in HUVECs

**Fig. 7:**
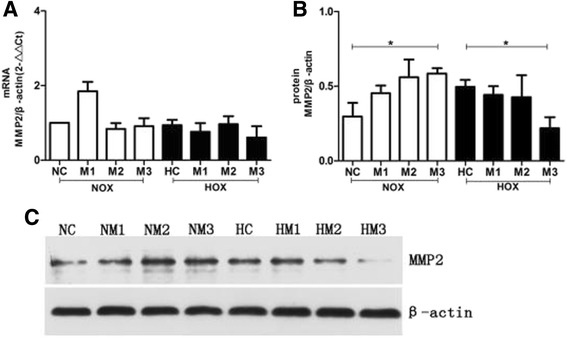
Expression of MMP 2 in HUVECs with r-maspin. **a** The level of MMP 2 mRNA in HUVECs treated with r-maspin during normoxia and hypoxia. The mRNA level was calculated after normalization against *β*-actin mRNA. **b** The level of MMP 2 protein in HUVECs treated with r-maspin. These data were expressed as a relative measure compared to *β-*actin and normalized to a control sample run on each gel. *P < 0.05. **c** Representative western blot showing the level of MMP2 protein in HUVECs treated with r-maspin. NM1 (NM2, NM3): under normoxia with the concentration of r-maspin at 10 ng/ml (10^2^ ng/ml,10^3^ ng/ml); HM1 (HM2, HM3): under hypoxia with the concentration of r-maspin at 10 ng/ml (10^2^ ng/ml,10^3^ ng/ml)

**Fig. 8:**
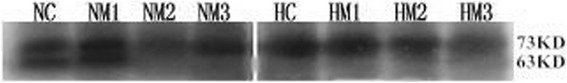
Gelatinolytic activity of MMP-2 by zymography in HUVECs treated with r-maspin during normoxia and hypoxia

Matrix metalloproteinases (MMPs) belong to the superfamily of calcium-dependent zinc endopeptidases and they are involved in tissue remodeling, angiogenesis and degradation of the extracellular matrix [18, 19]. No obvious change of expression level of MMP2 mRNA was observed in HUVECs that were treated with r-maspin under normoxia and hypoxia (Fig. 7a). However, the expression level of MMP2 protein increased with increasing concentration of r-maspin under normoxia while the change was only statistically significant at the highest concentration of r-maspin (Fig. 7b, c). The expression level of MMP2 protein was reduced with increasing concentration of r-maspin under hypoxia and the change was statistically significant at the highest concentration of r-maspin (Fig. 7b, c). Meantime, we could observe both non-active 72KD form and active 63KD form of MMP2 under normoxia while only non-active 72KD form of MMP2 was observed under hypoxia. We could only observe non-active 72KD form of MMP2 with high concentration of r-maspin under normoxia (Fig. 8).

## Discussion

It is generally agreed that PE originates from the placenta because the symptoms are quickly alleviated after its delivery [20]. The trophoblasts invade the wall of the uterus (interstitial invasion) and the uterine spiral arteries (endovascular invasion) gradually to create high-flow and low-resistance vessels [21]. Failure of vascular remodelling and widespread injury of endothelial cells contribute to the occurrence of preeclampsia. However, the exact molecular mechanism is unclear.

Previous work from our laboratory have found that maspin expression significantly was increased in preeclampsic placenta. Meantime, the expression of maspin was also significantly increased in TEV-1 followed by the decrease of TEV-1 invasion under hypoxia, which is in accordence with the physiology of preeclampsia. In this study, we found that maspin gene in HUVECs was expressed at a low mRNA level and maspin protein expression could not be detected. We have found that r-maspin with a concentration of 100 ng/mL significantly inhibited the aggressiveness of extravillous trophoblast cells (EVT) [22]. Here, we treated HUVECs with three different concentration of r-maspin. We observed that r-maspin significantly promoted the proliferation of HUVECs both under normoxia and hypoxia. However, it is generally believed that maspin plays an important role in tumor suppression by inhibiting tumor proliferation [23]. Machowska et al. [24] reported that the potential anti-proliferative activity of maspin is associated with its nuclear localization. They found a statistically significant negative relationship between the expression of nuclear maspin and Ki-67 in patients with invasive ductal breast cancer. Therefore we hypothesized that endogenous maspin and exogenous maspin have different effects on endothelial cells. Cella et al. [25] suggested that secreted maspin that derived from mammary epithelial acts on the cell surface to modulate cell adhesion.

Since there is only very slight expression of maspin in HUVECs both under normoxia and hypoxia, we consider whether exogenous maspin affect HUVECs function. No statistically significant change of apoptosis was observed in HUVECs that were treated with r-maspin. The result is consistent with the study that was reported by Li et al. [26], who also did not detect the expression of maspin and they have demonstrated that recombinant maspin did not increase HUVEC apoptosis regardless of dosage. Placental apoptosis is important for successful pregnancy [27] and abnormal apoptosis of trophblasts is involved in the pathogenesis of pre-eclampsia [28]. Apoptosis of vascular cells is also observed in normal vessel development in vivo [29]. Sandra et al. [21] suggests that uterine spiral artery remodeling involves endothelial apoptosis induced by extravillous trophoblasts through Fas/FasL interactions.

Angiogenesis is required for normal placental development. The pathogenesis of PE starts with dysfunction of trophoblast invasion and follows by impaired neovascularization of the placenta [3]. Therefore, Roberts et al. [30] suggested that PE is mainly caused by the dysfunction of vascular endothelial cells. The scratch assay and the tube formation assay were used for evaluating the migration and angiogenic ability [31] of vascular endothelial cells respectively. We observed that r-maspin had no effect on HUVECs while the ability of tube formation was significantly inhibited with r-maspin. Since Zhang et al. [8] have first demonstrated that maspin can effectively block neovascularization, a large number of studies have confirmed the inhibitory effect of maspin on tumor vessels [32–34]. And our results showed that r-maspin inhibited tube formation both under normoxia and hypoxia. Hence, narrow tube formation causes increased resistance of blood flow through the tube, which is consistent with the pathological characteristics of PE [4, 20]. In the same field of view, increased stenosis lumen required more endothelial to support its vessel-like structure, which is in accordance with the results of maspin in promoting cell proliferation. Meantime, in diffusion villi from PE placentas, increased vessel formation has been proposed to facilitate oxygen/nutrient transfer between the mother and fetus [35].

In addition to increased resistance, generalized vasoconstriction causes reduced utero-placental blood flow [36]. NO derived from endothelial cells is a vasorelaxant and anticoagulant factor [37]. Baker et al. [38] detected NO production and NO synthase activity in endothelial cells that were exposed to PE plasma, implying that endothelium-derived NO dysfunction is a potential cause of PE. Our results showed that r-maspin significantly inhibited NO synthesis. Therefore, maspin probably cause endothelial dysfunction by inhibiting NO synthesis.

Collectively, all the results implied that maspin inhibited angiogenesis both upon normoxia and hypoxia through promoting cell proliferation to compensate the need of increased tubes and on the other side impairing NO synthesis and thus caused increased resistance and generalized vasoconstriction.

MMPs contribute to the successful invasion of trophoblasts into spiral arteries by degrading the extracellular matrix [39]. MMP2 and MMP9 are the two main family members that participate in vascular remodeling [40]. The expression of MMPs in PE remains debatable. Zhu et al. [41] reported that the expression of MMP-2, −8, −9, and −11 was down-regulated in villous tissues of PE while Michal [42] and Galewska [40] reported the opposite results. Our results showed that the mRNA expression of MMP2 increased without statistical significance upon hypoxia compared with normoxia. We also observed that the level of MMP2 protein increased gradually during normoxia but decreased gradually upon hypoxia with increasing concentration of r-maspin. Meantime, r-maspin obviously inhibited MMP2 activity, which is consistent with the observation in the scratch assay. MMPs are a double-edged sword that can not only promote angiogenesis by combining pro-angiogenic factors such as VEGF [43] but also block angiogenesis by interacting with different MMPs such as MMP-12 and MMP-7 [43]. Thus, r-maspin may cause changes of MMP2 expression by combining different molecules during normoxia and hypoxia.

However, there were some limitations in our research. We used CoCl_2_ to induce chemical hypoxia. Though published studies have reported this method [13, 15], further research is needed to validate our research through the use of different concentration of oxygen to simulate hypoxia.

## Conclusions

Our results show that r-maspin causes endothelial dysfunction such as proliferation, tube formation, NO synthesis and MMP2 expression. Maspin may be an important participant for mediating endothelial function and ultimately lead to failure of the establishment of maternal-fetal blood circulation.

## Abbreviations

CoCl_2_: Cobalt dichloride
EVT: Extravillous trophoblast cells
FBS: Fetal bovine serum
HUVECs: Human umbilical vein endothelial cells
M1: 10 ng/ml
M2: 10^2^ ng/ml
M3: 10^3^ ng/ml
maspin: Mammary serine protease inhibitor
MMP: Matrix metalloproteinase
NO: Nitride oxide
OD: Optical density values
PE: Preeclampsia
PVDF: Polyvinylidene fluoride
r-maspin: Recombinant human maspin protein
serpin: Serine protease inhibitor
TEV-1: Human first-trimester extravillous trophoblast cell line
